# Attachment-related anxiety is associated with poor genital satisfaction and sexual problems in women

**DOI:** 10.1186/s12905-020-01110-6

**Published:** 2020-11-30

**Authors:** Nikola Komlenac, Margarethe Hochleitner

**Affiliations:** 1 Gender Medicine Unit, Medical University of Innsbruck, Fritz-Pregl-Strasse 3, 6020 Innsbruck, Austria; 2 Gender Medicine Unit, Medical University of Innsbruck, Innrain 66, 6020 Innsbruck, Austria

**Keywords:** Relational and Bodily Experiences Theory (RBET), Female sexual desire, Attachment, Genital satisfaction, Female sexual problems, Austria

## Abstract

**Background:**

Genital satisfaction has been found to influence women’s sexual experience. We tested the Relational Bodily Experiences Theory (RBET) that predicts associations between women’s genital satisfaction, attachment models, and sexual desire. We extended the model by additionally considering sexual arousal, orgasmic sensation, or the experience of pain during sexual activity as outcome variables. According to the RBET, women’s attachment models are associated with their genital satisfaction and linked to women’s sexual experience.

**Methods:**

A cross-sectional online questionnaire study was conducted at an Austrian medical university. In total 294 women (*M*_age_ = 23.7, *SD* = 3.4) provided full responses. Women were asked about genital satisfaction and experiences of distressing sexual problems. Attachment-related anxiety and avoidance were assessed with the Experiences in Close Relationships-Relationship Structures Questionnaire.

**Results:**

Results partially support the RBET. Attachment-related anxiety was associated with genital satisfaction which, in turn, was linked to experiences of frequent and/or distressing diminished sexual arousal, diminished sexual desire, or pain during sexual activity.

**Conclusions:**

These results suggest that clinicians should assess genital satisfaction when treating female sexual problems. Women with attachment-related anxiety may especially be prone to having poor genital satisfaction and may profit from body image interventions in order to improve their sexual experience.

## Background

The Relational Bodily Experiences Theory (RBET) [1] explains the inhibition or heightening of sexual desire in women by using the smallest number of relevant interpersonal and psychological constructs [2]. A previous study [3] showed that in accordance with the RBET, women’s attachment models are associated with their sexual body self-representation and sexual body self-representation, in turn, is linked to women’s sexual desire [1]. However, previous research reports that the two components of the RBET are associated not only with low sexual desire but also with other sexual dysfunctions [4–7]. Sexual dysfunctions in women include distressing experiences of low sexual desire, but also lack of sexual arousal, difficulties in attaining orgasm, and pain during sexual activity [8, 9]. A recent population-based study in Belgium reports the prevalence of distressing low sexual desire to be 4.9%, of distressing lack of sexual arousal 6.3%, of distressing difficulties in attaining orgasm 4.4%, and of pain during sexual activity 1.5% [10]. In order to consider all sexual dysfunctions, we tested and extended the RBET by including lack of sexual arousal, difficulties in attaining orgasm, and pain during sexual activity in addition to low sexual desire as outcome variables of the model.

Attachment models include peoples’ expectations of the availability, responsiveness, approval, or affection of an important (intimate) other person [11, 12]. People who have secure attachment models are confident that the other person is available or responsive and that the important person will always show approval or affection. On the other hand, people who have anxious attachment models are characterized by a lack of such confidence. People with avoidant attachment models have pessimistic views of relationships and avoid intimacy and emotional commitment [11]. Even though first attachment models are formed during infancy based on the availability and responsiveness of an important caretaker [11, 12], those early attachment models do not determine people’s attachment models in adulthood [13]. Additionally, by adulthood people often report strongest attachment to partners as compared to friends, siblings or parents [14]. Those attachment models for partners held in adulthood may differ from parent–child attachment models formed during infancy [13, 15–19]. In order to better guide therapy and interventions, Nichols [19] furthermore argued that it would be more informative to focus on current attachment models and their associations with women’s sexual functioning than to study parent–child attachment models of adult women. This is why in the current study we focused on attachment models of current or past intimate relationships with potential sexual partners and did not assess attachment models of parent–child relationships, as was done in a previous study [3].

According to the RBET, women’s attachment models are associated with their sexual body self-representation [1]. Sexual body self-representation includes the following concepts: sexual subjectivity (i.e. in how far a person regards themselves as a sexual being, and their sense of entitlement to sexual desire and pleasure [20]), self-objectification (i.e. a person’s tendency to take an observer’s perspective about one’s own body and judge oneself on the basis of whether the body fulfills social (heteronormative) expectations [21]), and genital self-image. Genital self-image refers to a person’s perceptions, thoughts and feelings about their genitalia. Positive evaluations of one’s genitalia result in genital satisfaction [22]. The Object of Desire Self-Consciousness Theory [23] gives a rationale for the relationship between attachment models and women’s genital satisfaction. According to the Object of Desire Self-Consciousness Theory [23], the assumption of not being romantically and sexually desirable to potential sexual partners is associated with poor body image. This is why especially women with attachment-related anxiety, who are often characterized as being fearful, feeling insecure about being a desirable partner or having negative self-appraisals or having low self-esteem [11, 24], may develop poor body image, including poor genital self-image. It was found that poor genital self-image is positively associated with low sexual desire, lack of sexual arousal, difficulties in attaining orgasm, and pain during sexual activity [4, 5, 25, 26]. In the current study, genital satisfaction was considered, because of the salience of genital satisfaction with regard to women’s sexual activity and female sexual pleasure [27].

In summary, the RBET has been supported by past research [3] and highlights the importance of attachment models and women’s sexual body self-representation in understanding women’s problems with sexual desire [1]. Therefore, attachment-based interventions and body image interventions [28] may be considered when treating low sexual desire in women. However, components of the RBET are associated not only with low sexual desire but also with other sexual dysfunctions [4–7], and clinicians may need to consider women’s attachment models and their sexual body self-representation not only when treating women with low sexual desire, but also with other sexual dysfunctions. The current study is one of the rare studies to analyze the associations between female attachment models, genital satisfaction and sexual dysfunctions [29]. The aim of the study was to empirically test and extend the RBET by considering all sexual dysfunctions [8, 9], including low sexual desire, lack of sexual arousal, difficulties in attaining orgasm, and pain during sexual activity as outcome variables.

## Methods

### Procedure and measures

The current study was part of a larger study regarding young adult pornography consumption and sexual health. This online questionnaire study was conducted at an Austrian medical university. All medical students at this medical university were contacted by e-mail and invited to participate in the study that was hosted on SoSci: der onlineFragebogen (https://soscisurvey.de/). Participants provided informed consent before accessing the questionnaire. Participation was voluntary, anonymous and all participants were able to withdraw from participation at any time. Authors’ contact information was provided on every page of the online questionnaire and participants were able to contact the authors for any questions concerning the study. The medical university’s Ethics Committee exempted the current study from full ethics review.

The first part of the questionnaire contained questions about sociodemographic variables. Participants were asked to self-report their gender (“*woman*”, “*man*”, “*other*”), their age, their sexual orientation (“*heterosexual*”, “*gay-identified/lesbian-identified*”, “*bisexual*”, “*asexual*”, “*other*”), their nationality (“*Austrian*” vs. “*German*” vs. “*Turkish*” vs. “*Italian*” vs. “*other*”) and their relationship status (“*single*” vs. “*in relationship*”). For any participant who chose “*other*” as a response an additional open text field allowed the response to be specified. The current analysis included only female participants. Because only a small percentage (2.0%) identified as lesbian, a dichotomous variable was formed for sexual orientation. Lesbian-identified and bisexual women (12.9%) were grouped in one category of the newly formed dummy variable for sexual orientation (non-heterosexual identified). Heterosexual-identified women (85.0%) were grouped in the other category.

Distressing sexual problems were assessed with two questions [30]. First, participants were asked how often (1 = *never*, 2 = *sometimes*, 3 = *often*, 4 = *always*) they had experienced each of the following sexual problems in the previous 6 months: pain during sexual activity, diminished sexual desire, diminished sexual arousal, or diminished intensity of orgasmic sensations. If prevalent, participants were asked how much distress (1 = *no distress*, 2 = *a little*, 3 = *considerable*, 4 = *much distress*) they felt in connection with each sexual problem. Responses to these two questions were multiplied to form one (continuous) variable for each distressing sexual problem, so that the variable contains information about the frequency of a sexual problem and the distress caused by such a problem [8, 10, 31, 32].

To assess women’s satisfaction with their genitalia an item based on The Body Parts Satisfaction Scale—Revised [33] or The Female Genital Self‐Image Scale [5] was formulated. Women were asked, “How satisfied are you with the appearance of your genitalia?” Participants indicated their satisfaction with this body part on a six-point Likert scale (1 = *not at all*, 6 = *totally satisfied*).

The Experiences in Close Relationships-Relationship Structures Questionnaire (ECR-RS) assesses attachment-related anxiety (Cronbach’s α = 0.83–0.91; three items) and attachment-related avoidance (Cronbach’s α = 0.81–0.87; six items) [34]. Responses were given on a 7-point Likert scale (1 = *low attachment-related anxiety or avoidance*; 7 = *high attachment-related anxiety or avoidance*). Only participants who responded to this part of the questionnaire with regard to their current or past partners were considered in the current study. In the current study the attachment-related anxiety scale achieved a reliability of Cronbach’s α = 0.85 (three items). Two items (Item 5 and Item 6) from the attachment-related avoidance scale were removed because of low loadings (λ < 0.5) on the factor for attachment-related avoidance in the structural equation model. The reliability of the shortened attachment-related avoidance scale was Cronbach’s α = 0.87 (four items).

### Statistical analysis

Descriptive statistics concerning participants’ responses included the percentages and means (standard deviations) of given responses. Spearman correlations between the variables that were included in the subsequent structural equation model (SEM) were calculated. For those analyses the Statistical Package for the Social Sciences (SPSS) for Windows, version 25.0 (IBM Corp., Armonk, NY, USA) was used.

**Fig. 1:**
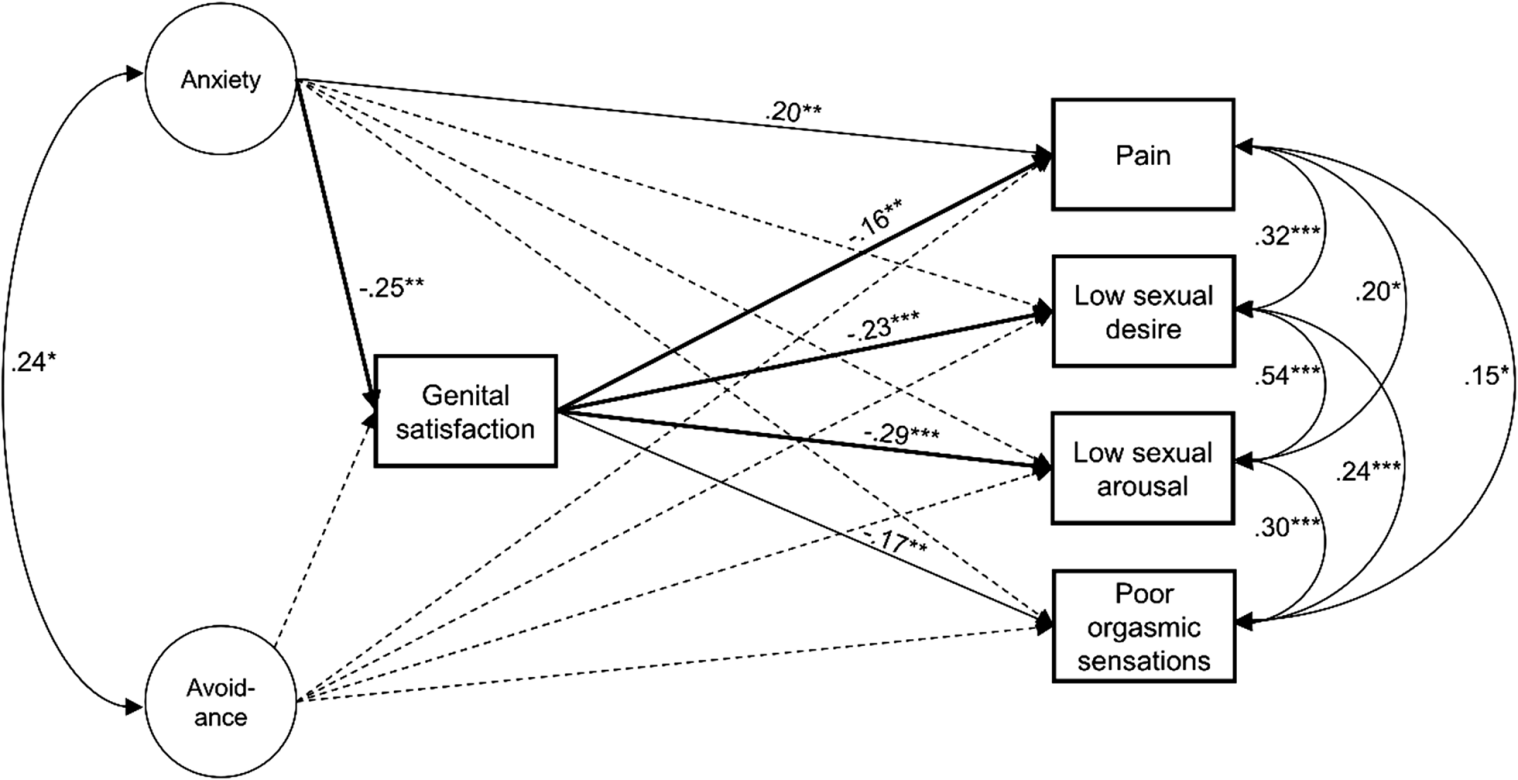
Structural equation model testing the Relational and Bodily Experience Theory. *Note* The structural equation model tested the Relational and Bodily Experience Theory. In the model the association between attachment-related anxiety or avoidance and sexual problems is estimated with mediating effects of genital satisfaction. The model was controlled for the sociodemographic variables age, sexual orientation and relationship status. Dotted lines represent non-significant relations; bold lines represent significant indirect paths. *p ≤ 0.05, **p ≤ 0.01, ***p ≤ 0.001

The SEM tested the relationships between attachment-related anxiety or avoidance, genital satisfaction and distressing sexual problems (Fig. 1). In the model each distressing sexual problem was predicted by genital satisfaction and attachment-related anxiety or avoidance. Furthermore, paths were calculated between attachment-related anxiety or avoidance and genital satisfaction. Indirect effects of attachment-related anxiety or avoidance on distressing sexual problems through genital satisfaction were estimated. Confidence intervals for the indirect model parameters were estimated with the Bollen–Stine bootstrap method using 500 bootstrap samples [35, 36]. The model was controlled for the sociodemographic variables age, sexual orientation and relationship status. The SEM was calculated using MPlus, Version 8 [36] (Muthén & Muthén, Los Angeles, CA, USA).

The mean-adjusted chi-square test statistic was used to determine model fit [36, 37], because variables violated the assumption of normal distribution (values of skew ranged from − 1.02 to 2.59 and values of kurtosis ranged from 0.38 to 9.16) [38]. A good model fit with the data was assumed when the following conditions were met: *p* values ≤ 0.05, ratio between chi-square statistics and respective degrees of freedom (χ^2^/*df*) ≤ 3.0 [39], root mean square error of approximation (RMSEA) ≤ 0.08 [40], standardized root mean square residual (SRMR) ≤ 0.10, comparative fit index (CFI) ≥ 0.90 [39]. The level of significance for all analyses was α = 0.05.

## Results

### Participants

In total 419 female participants took part in the online questionnaire study. Of the respondents, 115 were excluded because they reported their attachment with regard to friends or parental figures. Additional ten respondents were excluded from the analysis because they did not provide full responses to all questions relevant to the study. Finally, responses from 294 women (*M*_age_ = 23.7, *SD* = 3.4; range = 18–46 years) were included in the analysis. Most women (59.2%) indicated having Austrian nationality. Additional nationalities reported were: German (21.1%), Italian (16.3%), or other (3.4%). Most women (85.0%) self-reported having a heterosexual sexual orientation. The majority (77.2%) of the participants were in a relationship at the time of the study.

### Descriptive statistics

Most women reported little attachment-related anxiety or avoidance. The majority of women reported being satisfied with their genitalia (Table 1). With regard to diminished sexual desire, 44.9% of the participants had never experienced such difficulties. Of the participants 56.1% reported that they had not experienced pain during sexual activity in the previous 6 months. In the previous 6 months 66.7% of the participants had not experienced diminished sexual arousal, whereas 48.6% had not experienced diminished intensity of orgasmic sensations. The means of the composite variables of the distressing sexual problems (Table 1) indicate that 4.8% of the women experienced frequent and/or distressing pain during sexual activity (composite value ≥ 8). With regard to diminished sexual desire, 7.8% of women experienced such a difficulty frequently and/or were considerably distressed by it. In the previous 6 months 3.8% of the participants had frequent and/or distressing experiences of diminished sexual arousal, whereas 8.6% had frequent and/or distressing experiences of diminished intensity of orgasmic sensations.

**Table 1:** Means and Spearman correlations (n = 294)

	*M* (*SD*); range	2	3	4	5	6	7	8	9	10	11
1. Age	23.7 (3.4); 18–46	0.12*	0.08	0.02	− 0.03	0.08	0.13*	− 0.13*	− 0.08	− 0.07	− 0.04
2. Nationality^a^			− 0.13*	− 0.01	0.02	0.07	0.00	− 0.08	− 0.04	− 0.02	0.05
3. Relationship status^b^				0.02	− 0.33**	− 0.33**	0.01	0.02	0.16**	0.14*	− 0.03
4. Sexual orientation^c^					− 0.07	− 0.03	0.01	0.01	0.07	0.15**	− 0.03
5. Anxiety	2.5 (1.5); 1–7					0.45**	− 0.19**	0.11	− 0.01	0.02	0.16**
6. Avoidance	1.6 (0.9); 1–7						− 0.17**	0.06	0.06	0.07	0.22**
7. Genital satisfaction	4.7 (1.2); 1–6							− 0.19**	− 0.25**	− 0.22**	− 0.22**
8. Pain	2.5 (2.4); 1–16								0.33**	0.26**	0.12*
9. Low sexual desire	3.2 (2.8); 1–16									0.44**	0.31**
10. Low sexual arousal	2.1 (2.0); 1–12										0.30**
11. Poor orgasmic sensations	2.8 (2.7); 1–16										

^a^The baseline was Austrian = 1 (German = 2, Italian = 3, other = 4)

^b^The baseline was single = 1 (in relationship = 2)

^c^The baseline was heterosexual-identified = 1 (non-heterosexual-identified = 2)

*p ≤ 0.05, **p ≤ 0.01

Bivariate correlations between variables are reported in Table 1. Attachment-related anxiety and avoidance were negatively correlated with genital satisfaction. Women who reported poor genital satisfaction were more likely to report frequent and/or distressing experiences of each sexual problem studied. There was a positive association between attachment-related anxiety or avoidance and the experienced diminished intensity of orgasmic sensations.

### Testing the Relational and Bodily Experiences Theory

The structural equation model proved to have a good fit with the data, χ^2^(53) = 149.6, *p* < 0.001; χ^2^/*df* = 2.8; RMSEA = 0.079, 90%CI [0.064–0.094]; CFI = 0.937; SRMR = 0.051. All path coefficients of direct effects are reported in Table 2. While controlling for age, sexual orientation and relationship status the negative association between attachment-related anxiety and genital satisfaction remained significant (Fig. 1). On the other hand, attachment-related avoidance was not associated with genital satisfaction or with any sexual problem when controlling for age, sexual orientation and relationship status. Genital satisfaction was linked to women’s experiences of sexual problems. Women with poor genital satisfaction were more likely to experience frequent and/or distressing experiences of diminished sexual desire, diminished sexual arousal, diminished intensity of orgasmic sensations, or pain during sexual activity (Fig. 1). In addition, attachment-related anxiety was positively associated with the experience of frequent and/or distressing experiences of pain during sexual activity (Fig. 1). In total, the model explained 11% of variance of the experiences of diminished sexual desire, 19% of variance of diminished sexual arousal, 6% of variance of diminished intensity of orgasmic sensations, and 9% of variance of pain during sexual activity. The analyses of indirect effects were performed only to analyze the indirect effect of attachment-related anxiety on the sexual problems through genital satisfaction, because of the non-significant associations between attachment-related avoidance and the other variables (Fig. 1). The analysis of indirect effects revealed that attachment-related anxiety had an indirect effect on diminished sexual desire, diminished sexual arousal, and pain during sexual activity through genital satisfaction (Table 3).

**Table 2:** Path coefficients of direct effects in the structural equation model testing the Relational and Bodily Experience Theory

Predictor variable	Outcome variable	*B*	*SE B*	β	95% CI for β	
					*LL*	*UL*
Attachment-related anxiety						− 0.11
	Gential satisfaction	− 0.22**	0.07	− 0.25**	− 0.38	
	Pain	0.36*	0.15	0.20**	0.08	0.32
	Low sexual desire	0.02	0.19	0.01	− 0.12	0.15
	Low sexual arousal	0.15	0.13	0.10	− 0.03	0.24
	Poor orgasmic sensations	0.20	0.15	0.10	− 0.02	0.22
Attachment-related avoidance						0.05
	Gential satisfaction	0.08	0.10	0.06	− 0.18	
	Pain	0.05	0.18	0.02	− 0.07	0.12
	Low sexual desire	0.16	0.22	0.05	− 0.04	0.16
	Low sexual arousal	0.19	0.15	0.08	− 0.01	0.19
	Poor orgasmic sensations	0.23	0.34	0.07	− 0.08	0.28
Gential satisfaction						− 0.06
	Pain	− 0.32*	0.13	− 0.16**	− 0.26	
	Low sexual desire	− 0.54***	0.15	− 0.23***	− 0.33	− 0.13
	Low sexual arousal	− 0.51***	0.13	− 0.29***	− 0.41	− 0.18
	Poor orgasmic sensations	− 0.40**	0.14	− 0.17**	− 0.28	− 0.07

χ^2^(53) = 149.6, *p* < 0.001; χ^2^/*df* = 2.8; RMSEA = 0.079, 90%CI [.064–.094]; CFI = 0.937; SRMR = 0.051; CI = confidence interval

^*^p ≤ 0.05; **p ≤ 0.01; ***p ≤ 0.001

**Table 3:** Indirect effects of attachment-related anxiety on sexual problems through genital satisfaction

Sexual problem	*B*	*SE B*	β	95% CI for β	
				*LL*	*UL*
Pain	0.07	0.04	0.04*	0.01	0.08
Low sexual desire	0.12*	0.05	0.06*	0.03	0.11
Low sexual arousal	0.11*	0.05	0.07*	0.03	0.13
Poor orgasmic sensations	0.09	0.05	0.04	0.01	0.09

χ^2^(53) = 149.6, *p* < 0.001; χ^2^/*df* = 2.8; RMSEA = 0.079, 90%CI [.064–.094]; CFI = 0.937; SRMR = 0.051; CI = confidence interval

^*^p ≤ 0.05

## Discussion

The current study adds partial support for the Relational Bodily Experience Theory (RBET) [1]. According to the RBET, women’s attachment models are associated with their sexual body self-representation. Sexual body self-representation, in turn is linked to women’s sexual desire [1]. The current study’s findings reveal that attachment-related anxiety is associated with genital satisfaction and is consequently linked to experiences of frequent and/or distressing diminished sexual desire. Our study extends previous findings [3] by extending the model. Our study adds the finding that women’s attachment models and genital satisfaction are not only related to sexual desire, but are also associated with the experience of sexual arousal or the experience of pain during sexual activity.

Our study’s results are in line with studies that found that attachment-related anxiety, in particular, is a strong predictor for body dissatisfaction [41], body surveillance, or body shame [42]. Women with attachment-related anxiety, who are afraid they would not receive approval or affection from an important intimate person [11], may also doubt that they are romantically and sexually desirable in another’s eyes. The Object of Desire Self-Consciousness Theory [23] explains that people who doubt that they are romantically and sexually desirable in another’s eyes may also develop poor body image. Poor body image, including poor genital satisfaction may lead to distracting thoughts about one’s genitalia during sexual activity. Such cognitive distraction often negatively impacts sexual activity [6, 43, 44]. In accordance, in the current study, poor genital satisfaction was associated with sexual problems, such as low sexual desire, experience of sexual arousal problems, experience of poor orgasmic sensation, or the experience of pain during sexual activity.

By extending the RBET the current study shows that attachment models and sexual body self-representation may not only be relevant when treating women with low sexual desire [1], but also with other sexual dysfunctions [8, 9], such as lack of sexual arousal, difficulties in attaining orgasm, and pain during sexual activity. Clinicians who treat women with sexual problems should address their clients’ or patients’ body satisfaction including genital satisfaction. Body image interventions [28] and especially those that help clients or patients prioritize body functionality over body aesthetics [45–47] may help women develop a positive body image and this may consequently improve their sexual experience. The RBET highlights that such body image interventions should also consider women’s attachment models because attachment models are associated with body dissatisfaction [41]. Emotionally focused therapies that emphasize attachment-related needs may be considered for women with attachment-related anxiety [48] because of the close association between attachment-related anxiety, genital satisfaction and sexual activity [49].

In our study, however, attachment-related anxiety and genital satisfaction could explain only small to medium proportions of variance for the experiences of frequent and/or distressing sexual problems, including sexual desire [50]. Even though the authors of the RBET tried to explain sexual desire in women by using the smallest number of relevant interpersonal and psychological constructs [2], many other interpersonal and psychological factors not included in the RBET have been found to be associated with distressing sexual problems [51, 52] or genital satisfaction [53]. Future studies should explore whether the model can be extended by factors such as internalization of gender role norms, exposure to peer influence, exposure to media, or depression or anxiety, all of which have been associated with distressing sexual problems or genital satisfaction [25, 51–53].

The current study tested only specific aspects of the RBET and did not include all concepts integrated in the model. For instance, we used only one aspect of the proposed concept for sexual body self-representation, namely genital satisfaction. We tested only genital satisfaction, because of the salience of genital satisfaction with regard to women’s sexual activity and female sexual pleasure [27]. Additionally, in a previous study that tested the RBET [3], not all of the hypnotized components of sexual body self-representation (i.e. sexual subjectivity, self-objectification, and genital satisfaction) had strong loadings on this one factor. Therefore, analyzing those components separately may be more informative and conclusive in future.

## Limitations

This study has its limitations. First, the current study cannot make any predictions or conclusions about the directionality or causality of found associations, because the results are based on a cross-sectional study. Future longitudinal studies or experimental studies could shed light into the directionality of found associations. Furthermore, the study was conducted in a convenience sample of medical students and results should therefore be interpreted with caution and not be overgeneralized. Second, we used only one self-constructed item for the assessment of genital satisfaction instead of a validated scale [5] in order to keep the questionnaire short and not risk discontinuation by participants before reaching the end of the questionnaire. However, the used item is similar to questions used in validated questionnaires [5, 33] and results should therefore be comparable. Nevertheless, future studies should refrain from using self-constructed items that were not subject to psychometric analysis. Last, as is the case with many questionnaire studies, the current study is based on self-reports and may have been biased by participants’ inaccurate responses. Some participants may have given inaccurate responses because of social desirability, they may have felt some questions to be intrusive, or they may have had problems understanding some questions on the questionnaire [54].

## Conclusions

In spite of the study’s limitations, this is one of the rare studies to test the association between female attachment models, genital satisfaction and sexual desire [29]. The associations that were theoretically predicted by the Relational Bodily Experience Theory (RBET) are partially supported by the current study’s results. Attachment-related anxiety, but not attachment-related avoidance, was associated with genital satisfaction. Genital satisfaction, in turn, was linked to experiences of frequent and/or distressing diminished sexual desire. The current study also shows that the factors that predicted sexual desire in the RBET may also influence other components of sexual activity, such as sexual arousal, the experience of orgasmic sensation or the experience of potential pain during sexual activity. It can be recommended that clinicians who treat women with sexual problems also assess their clients’ or patients’ body satisfaction including genital satisfaction. Body image interventions may be considered an important treatment option for female sexual problems, especially when treating women with attachment-related anxiety.

## Data Availability

The datasets used and/or analyzed during the current study are available from the corresponding author on reasonable request.

## Abbreviations

RBET: The Relational Bodily Experience Theory
ECR-RS: The Experiences in Close Relationships-Relationship Structures Questionnaire
SEM: Structural equation model
RMSEA: Root mean square error of approximation
SRMR: Standardized root mean square residual
CFI: Comparative fit index
